# Spatial distribution and determinants of stunting, wasting and underweight in children under-five in Ethiopia

**DOI:** 10.1186/s12889-023-15488-z

**Published:** 2023-04-04

**Authors:** Kenenisa Abdisa Kuse, Dereje Danbe Debeko

**Affiliations:** 1 College of Natural and Computational Science, Bule Hora University, Bule Hora, Ethiopia; 2 College of Natural and Computational Science, Hawassa University, Hawassa, Ethiopia

**Keywords:** Under-five children, Malnutrition, Spatial analysis, Ethiopia

## Abstract

**Background:**

The burden of malnutrition in children under five remains an important child health issue where significant regional variations are observed in Ethiopia. The main aim of the current study was to explore the spatial distribution of stunting, wasting, and underweight in children under five in Ethiopia.

**Methods:**

The secondary data from EDHS, 2016, was employed, and a total of 7960 under-five children were included in the analysis. The general spatial analysis was performed to explore the spatial distribution of malnutrition among under-five within and between the regions of Ethiopia. The Spatial Lag and Spatial Error models were used to examine the spatial dependence between stunting, wasting, and being underweight. The Geographically weighted regression analysis was performed to estimate the types and strength of relationships between malnutrition and associated determinant factors across the regions and within the clusters or Zones of each region.

**Results:**

Among the under-five children included in the study, 36.6% were stunted, 12.2% were wasted and 25.2% were underweight. The Global Moran Index's value indicates (Stunting, I = 0.3135, *p*-value < 0.00001, Wasting, I = 0.1948, *p*-value < 0.0001 and Underweight, I = 0.5291, *p*-value < 0.0001) that there was a significant spatial variation of malnutrition across the regions and Zones of Ethiopia. The significant source of spatial variation of malnutrition in children under five was associated with the mother's education level, drinking water facility, toilet facilities, number of children under-five in the household, household’s wealth index, breastfeeding duration of the child, child size at birth, Body Mass Index of Mothers (BMI), region, and place of residence.

**Conclusions:**

The spatial association and spatial patterns of stunting, wasting, and being underweight were clustered within and between the regions of Ethiopia. The hotspot areas of stunting, wasting, and being underweight were detected in the regions where there were poor health facilities and limited socioeconomic indicators.

**Supplementary Information:**

The online version contains supplementary material available at 10.1186/s12889-023-15488-z.

## Introduction

Malnutrition is a complex condition and caused by multiples and overlapping factors including poverty, low parental education, lack of sanitation, low food intake, diarrhea and other infections, poor feeding practices, family size, short birth intervals, maternal time availability, child-rearing practices, and seasonality. There are also economic, social, and cultural causes of malnutrition which underscore the close link between malnutrition [1]. Unbalanced nutrient intakes, a lack of essential nutrients, or impaired nutrient utilization refer to health and well-being deficiencies [2]. The nutritional well-being of infants and young children is a significant public health concern since their bodies require more nutrients such as vitamins and minerals [3].

Child stunting, wasting, and being underweight are the leading public health issues in developing countries, resulting in child morbidity and mortality [3]. Globally, there were 21.9%, 13.4%, and 7.3% of stunted, underweight, and wasted under-five children, respectively, [4]. The highest prevalence of child stunting, wasting, and underweight have been found in sub-Saharan Africa [5]. Among the estimated 5.4 million under-five deaths each year, 2.7 million deaths were accounted for in this region including Ethiopia [6]. Based on the Ethiopian Demographic and Health Survey (EDHS), 2016[7], among the under-five children included in the survey, 38% were stunted, 10% were wasted, and 24% were underweight. This figure has been shown promising drop for the past two decades. However, it still remains an under-five health problem ranking Ethiopia among the top malnutrition burdened countries in the world [7–9].

The tremendous efforts has been made to investigate and identify various determinant factors associated with malnutrition (stunting, wasting, and being underweight) in children under five in Ethiopia [10–12]. Most of the previous findings under taken in the country were based on the descriptive analysis using anthropometric measurements [13–15]. Researchers have tried to estimate whether there was a significant variation across the regions of Ethiopia using hierarchical models with only two-level variate are accounted [16–18]. To the best of our knowledge, no studies have been estimated spatial distribution of stunting, wasting, and underweight in children under-five accounting for regional and zonal level variations. The hot spot and cold spot area within the regions, geographically weighted spatial distributions, and spatial dependencies of malnutrition (stunting, wasting, and underweight) in children under-five were not yet estimated. Thus, the current study was aimed to explore the spatial distribution of stunting, wasting, and underweight in children under-five and to identify associated determinants.

## Data

The target population of this study was children under-five in Ethiopia. The study data was taken from the Ethiopia Demographic and Health Survey (EDHS, 2016) [7], conducted by the Central Statistical Agency of Ethiopia (www.dhsprogram.com). Survey was conducted based on stratification and selection stages. Each region was stratified into urban and rural areas yielding 21 sampling strata. The enumeration areas (EAs) were selected independently in each stratum in two stages. In the first stage, a total of 645 EAs (202 in urban areas and 443 in rural areas) were selected with probability proportional to EA size and with independent selection in each sampling stratum. In the second stage, a fixed number of 28 households per cluster were selected with an equal probability of systematic selection from the newly created households. A total of 10,641 children under-five were identified in the households of selected clusters [7].

Based on some exclusion criteria (children who were not alive during survey, had incomplete observations, had no complete anthropometric measurements and whose Z-scores fell outside WHO Child Growth Standards plausible range(height-for-age z-scores below -6 SD or above + 6 SD, weight-for-age z-scores below -6 SD or above + 5 SD, weight for height z-scores below -5 SD or above + 5 SD)) only 7960 under-five children were included in the analysis [2].

In the current study, there were three anthropometric indicators used to calculate types of malnutrition based on the standard score values (Z-score). The Z-scores indices for measuring nutritional status were calculated according to the WHO recommendations (Stunting: child’s height for age Z-scores < -2 SD of the median WHO reference values, Underweight: child’s weight for age Z-score is < -2 SD from the median WHO reference values and Wasting: child’s weight for height Z-score is < -2 SD from the median WHO reference values) [2].

2.1 $$Z\frac{{X}_{i}-\mu }{{\delta }_{r}}$$

where, ${X}_{i}$ = height or weight of a given child at age x, $\mu$ = mean or 50^th^ percentile of the reference population at age x, and ${\delta }_{r}$= standard deviation of the reference population at age x.

A total of 21 independent covariates and the Zonal level (third administrative level) variables were extracted from the Ethiopian shape file map (https://africaopendata.org/dataset*)* of spatial coding by the Central Statistical Agency of Ethiopia in 2016. There were a total of 68 sub-divided zones in the survey data (Supplementary Table) where Harari region, Dire Dawa, and Addis Ababa have no rural administrative zones.

### Statistical methods: spatial analysis

Spatial analysis is an analysis that includes the influence of spatial or space into the analysis and includes any of the formal techniques which study about topological, geometric, or geographic properties [19]. In the current study, the spatial analysis was used to explore the spatial distribution of stunting, wasting and underweight in children under-five across and with the regions of Ethiopia. The Hot Spot Analysis tool calculates the Getis-Ord Gi* statistic for each feature in a dataset. The resultant z-scores and *p*-values indicates where there was either high or low values in the spatial clusters that accounts each feature within the context of neighboring features [19].

### Spatial scan analysis

To identify whether there is the presence of the statistically significant spatial clusters for variables of interest spatial scan tests can be used [20]. Under-five children who were wasted, stunted, and underweighted were considered as cases, and children who had normal nutritional status were considered as a control to fit the proposed statistical models.

### Spatial dependence model

Spatial dependence refers to the degree of spatial autocorrelation between independently measured values observed in geographical space. In this study, the spatial dependencies were used to measure the degree of associative dependences between stunting, wasting, and underweight. The spatial regression models can be used when the outcome of interest were correlated with the outcomes of its neighbors (conditional on other variables). If there is no spatial dependency, the ordinary least squares (OLS) estimation method may be a better choice [21]. The two common types of spatial dependence models used in this study are spatial lag models and spatial error models.

### Spatial error model

Spatial error regression model is a model that takes into account the dependency of error values from neighboring areas associated with it. It emerges from the presence of spatial dependence in the error term of a spatial unit and the corresponding neighboring units. This modeling approach assumes the error terms across different spatial units to be correlated. However**,** using spatial error in OLS methods for spatial regression model parameter estimation, the assumption of uncorrelated error terms could be violated and the estimates could be inefficient [22]. In the current study, the spatial error model is used to account for spatial dependencies using an error term that could be associated with spatially lagged error terms on target groups.

A spatial error model [22]:

2.2 $$Y=\mathrm{X}\beta + \varphi ;and \varphi =pW+ \varepsilon$$

where, $Y$ is an n $\times$ 1 vector of the variable of interest (stunting, wasting and underweight), $X$ is an n $\times$ p design matrix of explanatory variables, $\beta$ is a p $\times$ 1 vector of regression coefficients, $\varphi$ is an n $\times$ 1 vector of error terms, $p$ is a scalar spatial error parameter, $\mathrm{W}$ is an n $\times$ n spatial weight matrix, and $\varepsilon$ is an n $\times$ 1 vector of error terms that are normally and independently distributes.

### Spatial lag model

Spatial lag regression model is a model that considers associated between areas and dependent variables. The mode is suggestive of a possible diffusion process – events in one area predict an increased likelihood of similar events in neighboring areas. This approach can be used to identify determinants associated with stunting, wasting, and underweight for i^th^ child in k^th^ region and j^th^ cluster (zones). In the current study, a spatial lag model assumes that there could be malnutrition dependencies among the clusters of interest [23].

A spatial Lag model [23]:

2.3 $$Y=X\beta +pWy+ \varepsilon$$

where, Y is an n $\times$ 1 vector of the malnutrition, $X$ is an n $\times$ p design matrix of explanatory variables, $B$ is a p $\times$ 1 vector of regression coefficients, *Wy* is the spatially lagged dependent variable for weights matrix *W*, ρ is spatial coefficient, *W* is an n $\times$ n spatial weight matrix, and $\varepsilon$ is an n $\times$ 1 vector of error terms. A positive value of ρ indicates that counties are expected to have higher rates of malnutrition if, on average, their neighbors have higher malnutrition.

### Spatial analysis: goodness of fit test

**Fig. 1:**
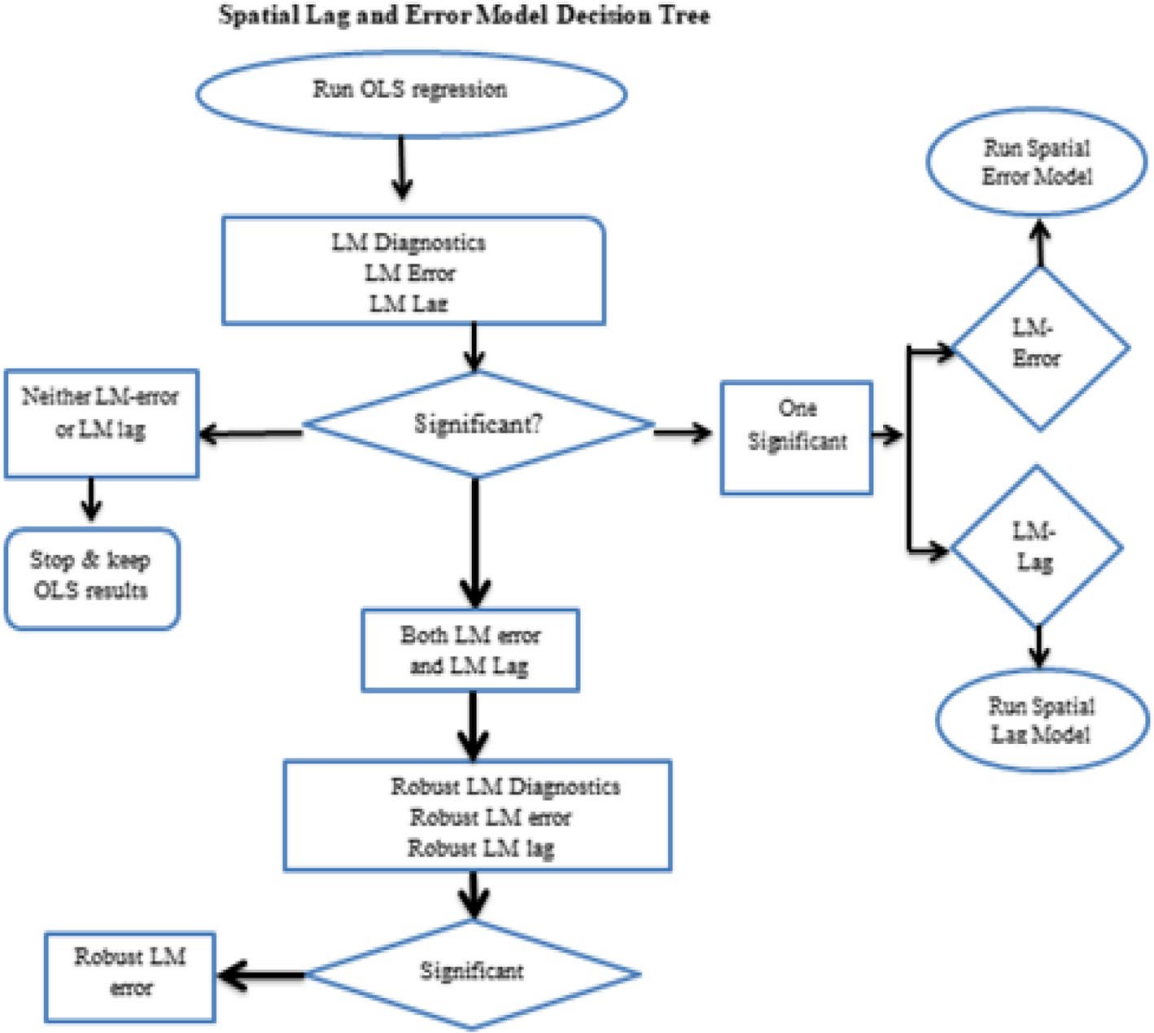
The decision tree of diagnostic procedures for spatial model selection. Source: Anseline, 2005 [25]

There are so many tests performed to assess the spatial dependence among the models. In the current study, the following test of spatial diagnosis has been used [24] as given in Fig. 1. To decide which model best fit the data, the Lagrange Multiplier (LM) test is important. The significance values of the LM-lag and Robust LM-lag tests suggest the validity of spatial lag model, while the significance values of the LM-error and Robust LM-error tests suggest the validity of spatial error model. The decision tree of spatial regression is shown in the diagnostic tree (Fig. 1).

### Parameter estimation

The most common method of estimating the spatial lag and spatial error model is the maximum likelihood estimation approach [26]. The point of departure is an assumption of normality for the error terms. The joint likelihood then follows from the multivariate normal distribution for the response variable. Unlike the classical regression model, the joint log-likelihood for a spatial lag or spatial error does not equal the sum of the log-likelihoods associated with the individual observations due to the two-directional nature of spatial dependence. The maximum likelihood estimate can be obtained from a numerical optimization of the concerned log-likelihood function [27]. The maximum likelihood estimation (MLE) method in this study is used to calculate the consistent and efficient estimate, and to better handle standard error for the regression parameter in the models used.

### Model comparisons

The aim of the model comparison is to identify model that best fits the data used for the current study. The most commonly used model selection criterions are Akaike’s Information Criterion (AIC) and Akaike’s Information Corrected Criterion (AICc). The AICc value can be used when sample size (n) is small and the rule of tamp is that the ratio of $\frac{n}{k}<40$ for the model with the largest number of parameters (k) examined. Any proposed model with small values of selection criteria are assumed to best fit the data used in the analysis. In the case of the large sample size, the value of AICc and AIC is the same and thus little is lost by being conservative and employing the information criterion for model diagnostics [28, 29].

### Spatial autocorrelation

The tests are used to show the amount or pattern of the spatial distribution of stunting, wasting, and underweight in Ethiopia. Two common measures of spatial autocorrelations considered in this study are Moran’s I and Gear’s C tests. The Moran's I is produced by standardizing the spatial auto covariance of the data. The Geary's c value uses the sum of the squared differences between pairs of data values as its measure of the variation [20]. In the current study, Moran’s I test is used to test whether there is spatial correlation of malnutrition between regions and within the Zones in each region.

### Spatial autocorrelation analysis

The expected value of Moran’s I is -1/(N-1). Values of I that exceed -1/(N-1) indicate positive spatial autocorrelation in which similar values either high or low values are spatially clustered. Values of I below -1/(N-1) indicate negative spatial autocorrelation indicating neighboring values are not similar. Theoretically, the expected value for Geary’s c is 1. The Geary’s c value less than 1 indicates positive spatial autocorrelation and a value larger than 1 point negative spatial autocorrelation between clusters [30].

2.4 $$I=\frac{N}{{S}_{0}}\frac{{\sum }_{i=1}^{N}\sum\limits_{j=1}^{N}{w}_{\left(i,j\right)}\left({x}_{i}-\overline{x }\right)\left({x}_{j}-\overline{x }\right)}{\sum\limits_{j=1}^{N}{w}_{\left(i,j\right)}\left({x}_{i}-\overline{x }\right)},i\ne j$$

2.5 $$c=\frac{(N-1)}{{2S}_{0}}\frac{{\sum }_{i=1}^{N}\sum\limits_{j=1}^{N}{w}_{\left(i,j\right)}{\left({x}_{i}-\overline{x }\right)}^{2}}{\sum\limits_{j=1}^{N}{w\left({x}_{i}-\overline{x }\right)}^{2}},i\ne j$$

where, N is the imputed values or the total values for neighboring features. Therefore, these study used 68 number zones or neighboring features, ${x}_{i}$ and ${x}_{j}$ are the observations for areas *i* and *j* with mean $\overline{\mathrm{X} }$ and $\overline{\mathrm{X} }$ is the mean of ${x}_{i}$, ${s}_{o}={\sum }_{I=1}^{N}\sum\limits_{j=1}^{N}{w}_{(i,j)}$, is the specified weighting scheme chosen. The variances of I and c will differ according to the model employed to the data. Under the randomization assumption used for Point Pattern Analysis (PPA), the variances of I and c can be calculated [31] as:

2.6 $$\mathrm{Var}\left(\mathrm{I}\right)=\frac{\mathrm{N}(({\mathrm{S}}_{1}\left({\mathrm{N}}^{2}-3\mathrm{N}+3\right)-({\mathrm{NS}}_{2}+3{{\mathrm{S}}_{0}}^{2})}{(\mathrm{N}-1)(\mathrm{N}-2)(\mathrm{N}-3){{\mathrm{S}}_{0}}^{2}}-\frac{\mathrm{K}({\mathrm{S}}_{1}\left({\mathrm{N}}^{2}-\mathrm{N}\right)-2{\mathrm{NS}}_{2}+6{{\mathrm{S}}_{0}}^{2}}{\left(\mathrm{N}-1\right)\left(\mathrm{N}-2\right)\left(\mathrm{N}-3\right){{\mathrm{S}}_{0}}^{2}}-{\left(\left.\frac{1}{\mathrm{N}-1}\right)\right.}^{2}$$

2.7 $$\mathrm{Var}\left(\mathrm{c}\right)=\frac{(\mathrm{N}-1){\mathrm{S}}_{1}({\mathrm{N}}^{2}-3\mathrm{N}+3-\mathrm{K}\left(\mathrm{N}-1\right))}{{{\mathrm{S}}_{0}}^{2}\mathrm{N}(\mathrm{N}-2)(\mathrm{N}-3)}+\frac{{({\mathrm{N}}^{2}-3-\mathrm{K}(\mathrm{N}-1)}^{2}}{\mathrm{N}(\mathrm{N}-2)(\mathrm{N}-3)}-\frac{(\mathrm{N}-1){\mathrm{S}}_{2}({\mathrm{N}}^{2}+3\mathrm{N}-6-\mathrm{K}({\mathrm{N}}^{2}-\mathrm{N}+2)}{4\mathrm{N}(\mathrm{N}-2)(\mathrm{N}-3){{\mathrm{S}}_{0}}^{2}}$$

Where I indicates Moran’s and c indicate Gear’s test value, and $${S}_{1,}$$$${S}_{2}$$, and K are expressed as follows:

$${\mathrm{S}}_{1}=\frac{1}{2}\sum_{\mathrm{I}=1}^{\mathrm{N}}\sum_{\mathrm{j}=1}^{\mathrm{N}}({\mathrm{w}\left(\mathrm{i},\mathrm{j}\right)+\mathrm{w}\left(\mathrm{j},\mathrm{i}\right))}^{2}$$

$${\mathrm{S}}_{2}=\frac{1}{2}\sum_{\mathrm{I}=1}^{\mathrm{N}}\sum_{\mathrm{j}=1}^{\mathrm{N}}({\mathrm{w}\left(\mathrm{i},\mathrm{j}\right)+\sum_{\mathrm{j}=1}^{\mathrm{N}}(\mathrm{w}(\mathrm{j},\mathrm{i})}^{2}$$

where,$\mathrm{w}\left(\mathrm{i},\mathrm{j}\right)$ is the element in the spatial weight matrix corresponding to the observation pair *i*, *j*; the two summations indicate total values in the N input by N matrix taken from the spatial weights matrix, and

$$K=\frac{N\sum_{i=1}^{N}{\left({x}_{i}-\overline{x }\right)}^{4}}{{\left(\sum_{i=1}^{N}({x}_{i}-\overline{x })\right)}^{2}}$$

### Geographically weighted regression model

Geographically weighted method is a modeling technique designed to handle non-stationary (the mean values vary by locations) spatial distributions. It has been widely used as a visualization tool to explore the patterns of spatial data. Geographical weighted regression model is used to locate the relationship between predictors and the variable of interest (stunting, wasting, and underweight). In the current study, this approach is used to explore the community-level factors and their effects on malnutrition at specific region and zones.

The geographically weighted regression model [31]:

2.8 $${Y}_{i}={\beta }_{o}\left({\upsilon }_{i}{v}_{i}\right)+\sum {\beta }_{o}\left(({\upsilon }_{i}{\nu }_{i}\right){X}_{ik}+\varepsilon$$

where, $${Y}_{i}$$ is the variable of interest, ($${u}_{i},{v}_{i})$$ are coordinates of the $${i}^{th}$$ point in space. $${\beta }_{0}({u}_{i},{v}_{i})$$ is a continuous functions of $$\beta$$, $${X}_{i1},\dots , {X}_{ik}$$ is the explanatory variables at point i, and $$\varepsilon$$ is an error.

## Results

### Descriptive statistics

From the total of 7960 under-five children, 38.4% of males and 34.8% of females were stunted, 13.5% of males and 10.9% of female were wasted, and 26.6% of males and 23.8% of females were underweight. Among the children aged from 6 to 11 months 19.1%, 17.9%, and 18% were stunted, wasted, and underweight, respectively. From the children aged between 12–23 months, 41.1%, 13.9%, and 26.2% were stunted, wasted, and underweight, respectively. From the children aged between 24–37 months, 47.4%, 13.9%, 28.4% were stunted, wasted, and underweight, respectively. Likewise, among children aged between 38—47 months, 46.4%, 17.9%, and 29.9% were stunted, wasted, and underweight, respectively., among children aged between 38—47 months 38.7%, 15.9%, 30.8% of them were stunted, wasted, and underweight, respectively (Table 1).

**Table 1:** Summary statistics of distribution of stunting, wasting, and underweight based on the indicator variable (EDHS, 2016)

Variables	Categories	Stunted N (%)	Wasted N (%)	Underweight N (%)
Child age in (months)	< 6	106 (10.5)	160 (10.8)	110(10.9)
	6–11	169 (19.1)	159 (8.1)	160 (18)
	12–23	642 (41.1)	217 (9.9	409 (26.2)
	24–37	882 (47.4)	184 (13.9)	528 (28.4)
	38–47	564 (46.4)	98 (17.9)	363 (29.9)
	48–59	553 (38.7)	154 (15.9)	439 (30.8)
Sex of child	Male	1548(38.4)	544(13.5)	1074(26.6)
	Female	1368(34.8)	428(10.9)	935(23.8)
	Total	2916(36.6)	972(12.2)	2009(25.3)
Place of residence	Urban	3456(76.1)	143(9.9)	194(13.5)
	Rural	2571(39.4)	829(12.7)	1815(27.8)
	Total	2916(36.6)	972(12.2)	2009(25.3)
Mothers educational level	No education	2068(40.4)	707(13.8)	1512(29.6)
	Primary education	693(33.9)	203(9.9)	400(19.6)
	Secondary education	112(21.5)	45(8.7)	65(12.5)
	Higher education	43(15.4)	17(6.1)	22(7.9)
	Total	2916(36.6)	972(12.2)	2009(25.3)
Source of drinking water	Improved Water	1717(35.1)	543(11.1)	1153(23.6)
	Un-Improved Water	1199(39.1)	429(14)	856(27.9)
	Total	2916(36.6)	972(12.2)	2009(25.3)
Type of toilet facility	Improved toilet	302(23.1)	121(9.3)	175(13.4)
	Un-improved toilet	2614(39.3)	851(12.8)	1834(27.6)
	Total	2916(36.6)	972(12.2)	2009(25.3)
Wealth index of households	Poorest	1163(40.4)	483(16.7)	940(32.6)
	Poorer	615(44.4)	157(11.3)	421(30.4)
	Middle	440(38.4)	127(11.1)	283(24.7)
	Richer	341(33.4)	86(8.4)	180(17.6)
	Richest	357(23.4)	119(7.8)	185(12.1)
	Total	2916(36.6)	972(12.2)	2009(25.3)
Fathers education level	No education	1598(41.3)	548(14.2)	1205(31.1)
	Primary	955(35.9)	268(10.1)	578(21.7)
	Secondary	235(29.5)	98(12.3)	150(18.8)
	Higher	128(20.3)	58(9.2)	76(12.1)
	Total	2916(36.6)	972(12.2)	2009(25.3)
Breastfeeding duration	Ever breastfeeding	1718(41.4)	430(10.4)	1155(27.9)
	Never breastfed	107(35.1)	37(12.2)	84(27.5)
	Still breastfeeding	1091(31.1)	505(14.4)	770(21.9)
	Total	2099(36.4)	972(12.2)	2009(25.3)

### Tests for spatial dependency

In regression analysis, sometimes, it is common to face problems that are inherently involved in geographic locations. In the current study the spatial lag model and spatial error model are used to estimate spatial dependencies in the different geographic clusters (Table 2).

**Table 2:** Spatial Dependency tests using the Lagrange Multiplier Diagnostics

Stunting									
Weights	Moran I statistic standard deviation		*P*-value	Alternative hypothesis	Observed Moran I		Expectation		Variance
bristol.W	41.154		< 3.3e-14	Greater	0.2715		-0.0054		0.0076
	LMlag, LMerror, and Robust LMlag, Robust LMeror Test for Stunting								
Weights	LMlag	*P*-value	LMerr	*p*-value	RMlag	*p*-value	RMerr		*p*-value
bristol.W	33.124	1.28e-11	694.1	3.3e-14	0.0182	0.5231	401.53,		1.1e-12
**Wasting**									
Weights	Moran I statistic standard deviation		*P*-value	Alternative hypothesis	Observed Moran I		Expectation		Variance
bristol.W	32.451		< 2.8e-15	Greater	0.3290		-0.0011		0.009
	**LMlag, LMerror, and Robust LMlag, Robust LMeror Test for Wasting**								
Weights	LMlag	*P*-value	LMerr		*p*-value	RMlag	*p*-value	RMerr	*p*-value
bristol.W	36.115	1.55e-11	523.6		< 2.8e-15	0.0216	0.6532	352.44	2.6e-14
**Underweight**									
Weights	Moran I statistic standard deviation		*P*-value	Alternative hypothesis	Observed Moran I		Expectation		Variance
bristol.W	58.33		< 2.44e-11	Greater	0.321		-0.001		0.008
	**LMlag, LMerror, and Robust LMlag, Robust LMeror Test for Underweight**								
Weights	LMlag	*P*-value	LMerr		*p*-value	RMlag	*p*-value	RMerr	*p*-value
bristol.W	39.121	3.11e-14	487.5		< 2.44e-11	0.0216	0.653	352.44	2.6e-14

Moran I test statistic for stunting, wasting, and underweight was 41.154, 32.45, and 58.33, respectively. The LMlag statistic for stunting, wasting, and underweight was 33.1, 36.115, and 39.121, respectively. The *p*-values of test statistic less than the level of significance ($\alpha =$0.05) for all cases indicates that both the spatial lag and the spatial error models better perform over the OLS model.

Since both models are significant in each response category. This indicates an additional test is required. Thus, the Robust LM test is used to identify tests which one could be at work. Here, we cannot reject the null hypothesis since there are spatial lags pointing to the possible presence of spatial errors in malnutrition (stunting, wasting, and underweight). Thus, for the three variables of interest, both simple tests of the lag and error models sign indicates the presence of spatial dependences of malnutrition across and within the regions of Ethiopia (Table 2).

### Model comparison

In this study, the test statistics clearly speak in favor of the spatial error model, but that may not always be the guarantee. A direct comparison between the three models has been done based on the maximized log-likelihood of the study parameters (Table 3).

**Table 3:** Model comparison using diagnostics test criteria

Stunting		Wasting		Underweight	
**Models**	**AICc**	**Models**	**AICc**	**Models**	**AICc**
OLS	335.676	OLS	294.926	OLS	411.516
SLM	254.516	SLM	330.336	SLM	258.316
SEM	164.426	SEM	197.316	SEM	200.616

Based on the model diagnostics analysis, the spatial error model has shown the best fit to the data than the other models proposed.

Age of child (in months), mothers educational level, source of drinking water, type of toilet facility, number of children under-five in the household, wealth index of households, sex of a child, breastfeeding duration, size of child at birth, and Body Mass Index of Mothers (BMI), region, and place of residence were a significant source of spatial variations of malnutrition in children under-five within and tween regions/zones of Ethiopia. The results from the spatial error model are described for each of the variables of interest (Stunting, wasting and underweight). Size of a child at birth had a significant association with stunting, wasting, and underweight ($\beta =.031,\mathrm{ CI}: .023-.039)$,($\beta =.012,\mathrm{ CI}: .0063- .017)$, and ($\beta =.028,\mathrm{ CI}: .021-.036)$ (Table 4).

**Table 4:** Parameter Estimation of stunting, wasting, and underweight using the spatial error model, EDHS, 2016

**Variables**	Stunting		Wasting		Underweight	
**Fixed Part**	Coef. (95% C.I)	*P*-value	Coef. (95% C.I)	*P*-value	Coef. (95% C.I)	*P*-value
Constant	.44 [.31-.57]	< 0.001	.28 [.19-.37]	< 0.001	.564 [.44-.68]	< 0.001
Child Age (in Months)	1.36 [1.02–1.44]	< 0.001	1.19 [1.12–2.45]	< 0.001	1.41 [1.29–1.99]	< 0.001
Mother education level	-.030 [-.04-(-.01)]	< 0.001	-.05 [-.02-(-.003)]	< 0.001	-.02[-.04-(-.007)]	< 0.001
Source of drinking Water	-.02 [-.04-(-.001)]	< 0.001	.306 [.19-.721]	< 0.001	-.030 [-.05-.009]	< 0.001
Type of toilet facility	.066 [.032-.1003]	< 0.001	.006 [.004-.0091]	< 0.001	.042 [.012-.072]	< 0.001
Number of under-five children in the household	-.111 [-.13-(-.08)]	< 0.001	.018 [.0009-.036]	< 0.001	-.07 [-.10-(-.05)]	< 0.001
Type of Cooking fuel	.062 [.013-.112]	. < 0.001	.024 [.019-.158]	< 0.001	.0010[-.043-.04]	.962
Wealth Index of households	-.01 [-.02-(-.009)]	< 0.001	-.019 [-.02-(-.01)]	< 0.001	-.033[-.04-(-.02)]	< 0.001
Age of Mothers at birth	-.012 [-.02-.005]	< 0.001	-.006 [-.01-.004]	.256	-.0094[-.02-.006]	.232
Fathers educational level	-.02 [-.03-(-.008)]	< 0.001	.005 [-.004-.015]	.257	-.01[-.03-(-.007)]	< 0.001
Mothers occupational status	.036 [.012-.059]	< 0.001	.0013 [-.014-.017]	.870	.022 [.001-.04]	.037
Birth Order Number	.013 [-.0019-.029]	.086	.0032 [-.007-.01]	.562	.0108 [-.003-.02]	.134
Sex of Child	-.038 [-.05-(-.01)]	< 0.001	-.029 [-.04-(-.01)]	< 0.001	-.03[-.051-(-.01)]	< 0.001
Preceding Birth Interval	-.048 [-.07-(-.02)]	< 0.001	-.014[-.031-.001]	.075	-.05 [-.07-(-.03)]	< 0.001
Breast feeding Duration	-.050 [-.06-(-.04)]	< 0.001	.022 [.015-.029]	< 0.001	-.02 [-.03-(-.01)]	< 0.001
Size of child at birth	.031 [.023-.039]	< 0.001	.012 [.0063-.017]	< 0.001	.028 [.021-.036]	< 0.001
BMI of Mothers	.081 [.069-.092]	< 0.001	-.082 [-.08-(-.07)]	< 0.001	-.100 [.11-(-.09)]	< 0.001
Region	-.35 [-.44-(-.27)]	< 0.001	-2.7 [-3.4-(-2.0)]	< 0.001	-.24 [-.33-(-.15)]	< 0.001
Place of residence	-.07 [-.08-(-.05)]	< 0.001	-.06 [-.08-(-.034)]	< 0.001	.36 [.12 -.60]	< 0.001
Sex of Household head	-.010 [-.037-.016]	.441	.012 [-.005-.031]	.175	.0012[-.022-.02]	.922
Current marital status of mother	.019 [-.040-.078]	.526	.0021 [-.038-.042]	.915	-.015 [-.06-.03]	.570

For a unit gain of weight at birth had 0.31, 0.12, and 0.28 times decreased prevalence.of being stunted, wasted, and underweight, respectively. The wealth index of mothers had a significant association with stunting, wasting, and underweight ($\beta =-.01 (95\mathrm{\%CI}: -.02,-.009)$, ($\beta =-.019 (95\mathrm{\%CI}:-.02, -.01)$, and ($\beta =-.033(95\mathrm{\%CI}:-.04, -.02)$. Mother with the better wealth index had shown 0.1, 0.19, and 0.33 times decreased prevalence of stunting, wasting, and underweight, respectively. Children from the households that had improved toilet facility had 0.66, 0.06, and 0.42 times decreased prevalence of stunting, wasting, and underweight (*β* = 0.066 (95%CI: 0.032, 0.1003), (*β* = 0.006 ($95\mathrm{\%CI}:$0.004, 0.0091), and (*β* = 0.042 ($95\mathrm{\%CI}:$0.012, 0.072), respectively (Table 4).

### Spatial autocorrelation for wasting, underweight, and stunting

**Fig. 2:**
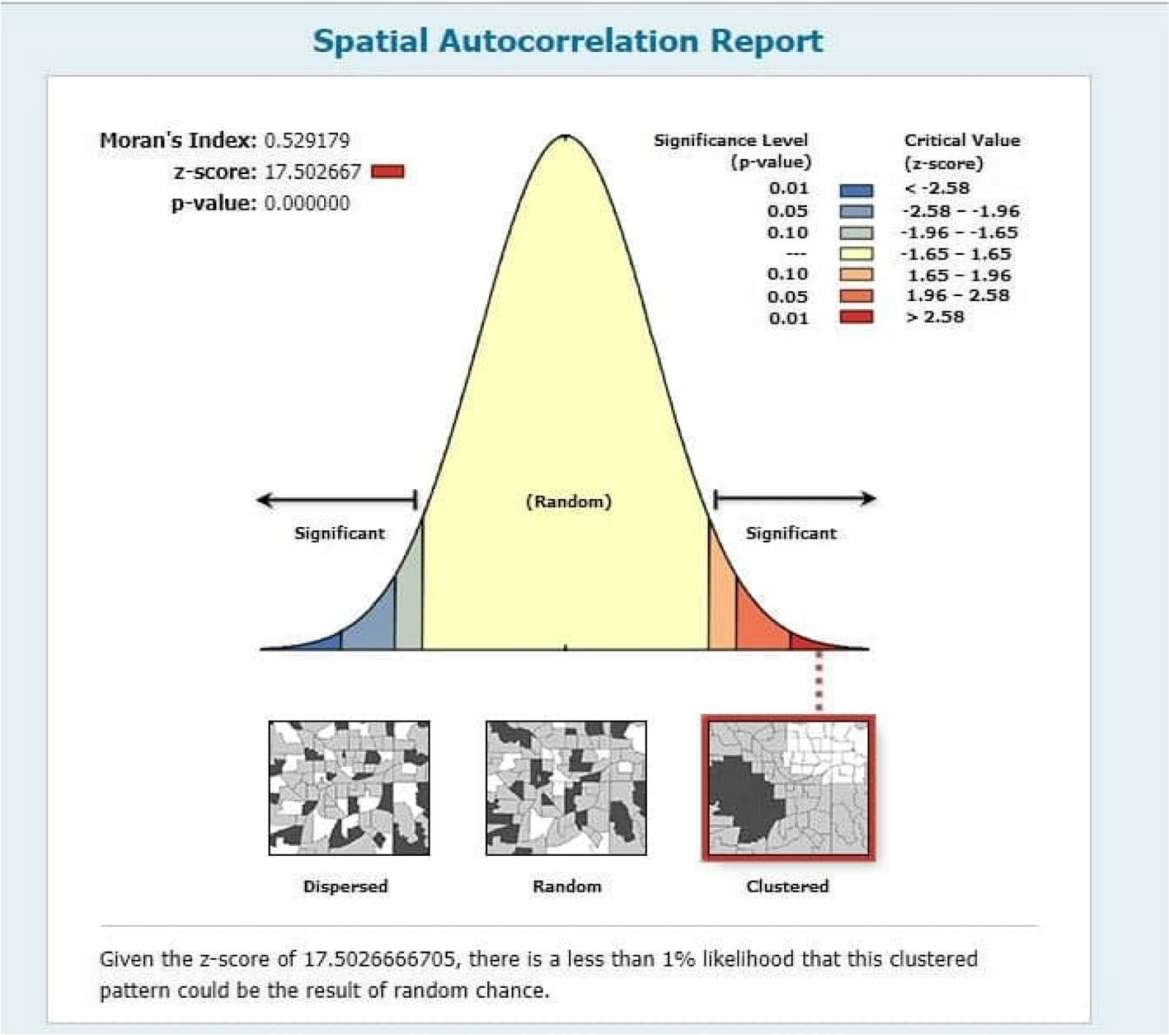
Spatial autocorrelation of underweight

**Fig. 3:**
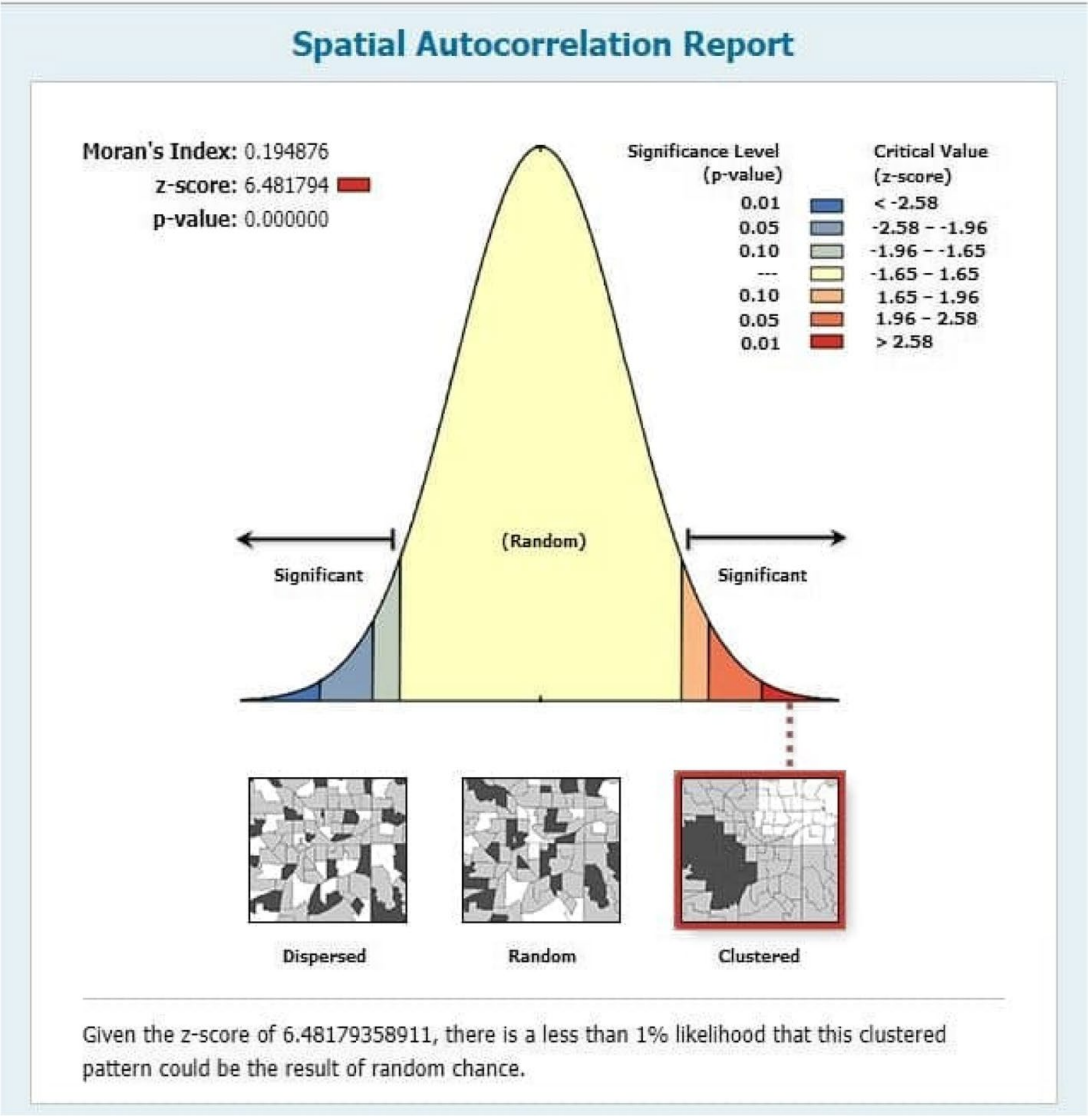
Spatial autocorrelation of wasting

**Fig. 4:**
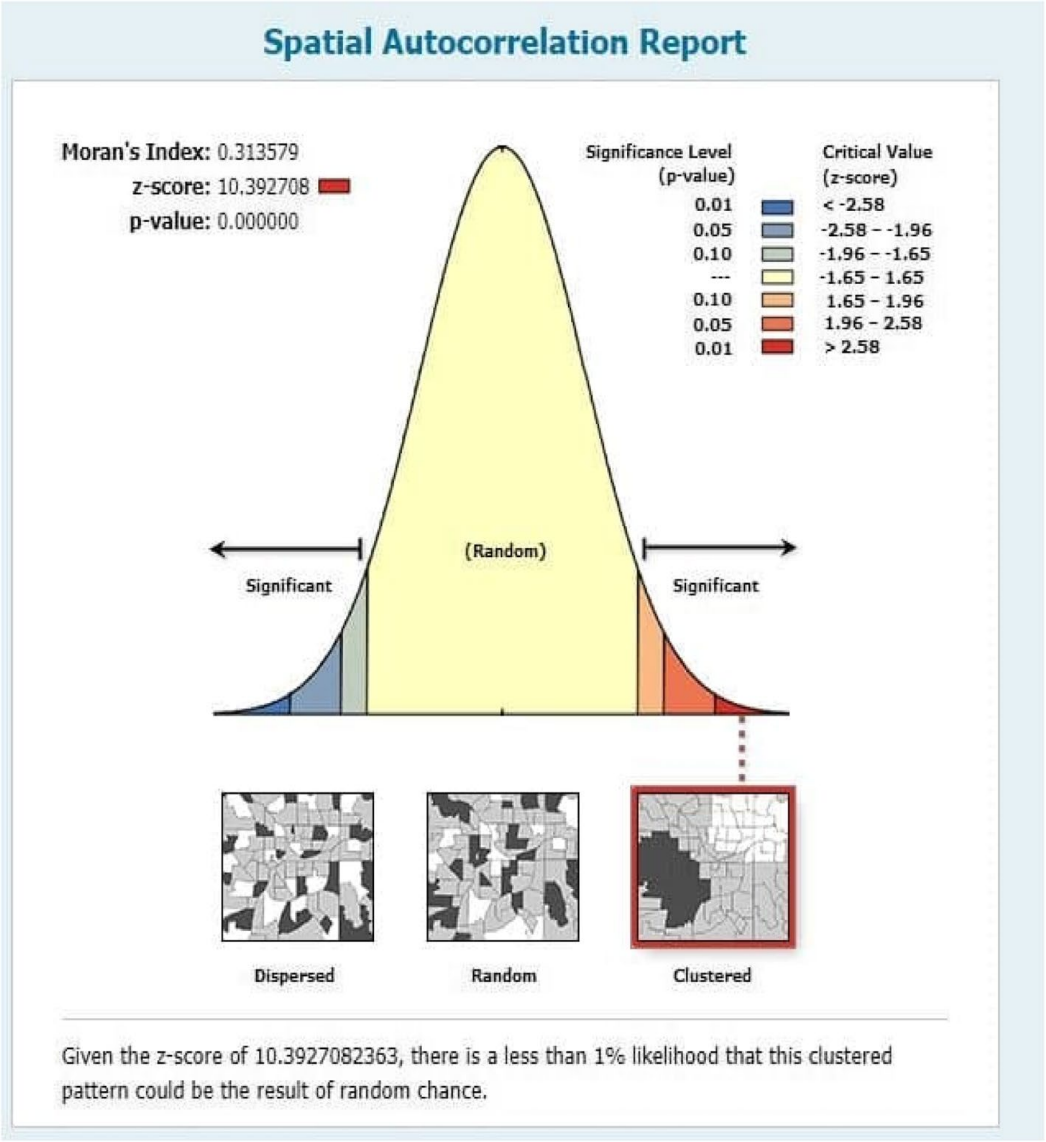
Spatial autocorrelation of stunting

The right side of each panel depicts a high rate of malnutrition in the study area for all the figures (Figs. 2, 3 and 4). Auto-generated interpretations displayed underneath each panel show that the likelihood of clustered patterns occurring by chance is less than 1%. The significant global clusters are indicated by the dark red color. The null hypothesis is rejected pointing that there was a significant difference in clustering of stunting, wasting and underweight, and the spatial distribution underlying random clusters since the *p*-value less than level of significance (*P* < 0.0001).

Spatial Autocorrelation: Underweight.

### Hotspots analysis

#### Stunting

**Fig. 5:**
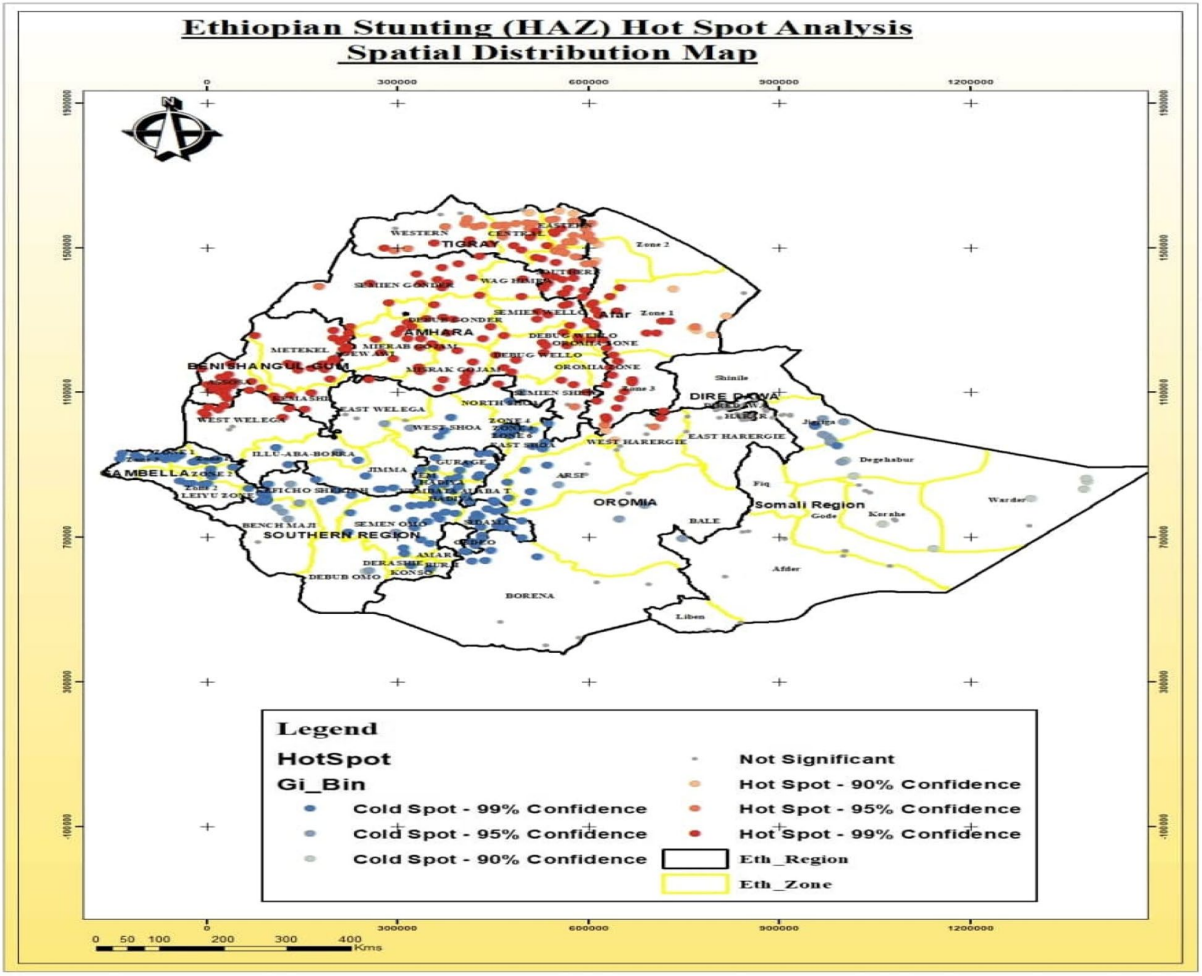
Spatial distribution of Hotspots and Cold spots areas of stunted among under-five in Ethiopia

The hotspot areas of stunting were detected (areas with higher rates) in the Amhara region, especially in West Gojjam Zone, Awi Zone, Wag Hemra Zone, and Benishangul-gumuz region specifically in Metekel Zone. The blue color indicates areas where there were significantly lower rates of stunting (cold spot areas) in Ethiopia. Some zones of the Oromia region (Arsi and East Shewa Zone), some parts of the Somali region (Jijiga zones), and the three zones of SNNPR (Kambata Tambaro, Hadiya, and Gedeo Zone) had significantly cold spots (lowest rate) distribution of stunting (Fig. 5).

#### Underweight

**Fig. 6:**
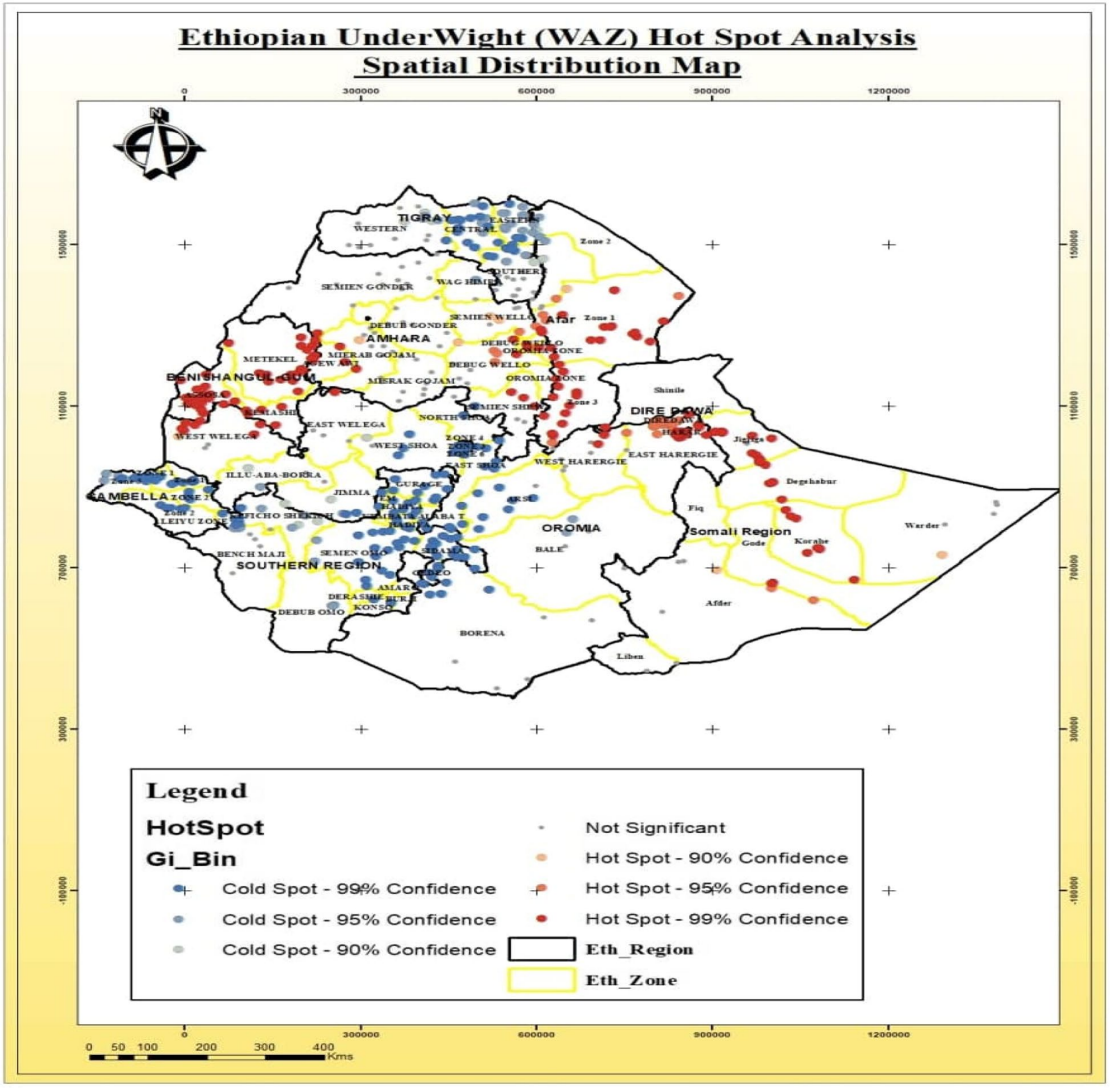
Spatial distribution of Hotspots and Cold spots areas of underweight among under-five in Ethiopia

The hotspot areas of underweight were detected in the Afar region (Awsi Zone), Benishangul-gumuz region (Assosa, and Kemashi Zone), and Amhara region (South Wollo, and Oromia-Special Zones). In contrast, the lower distribution rates (blue colors) of underweight (cold spot areas) were detected in the SNNPR region (Gurage, Hadiya, and Kambata Tambaro Zones), and Oromia region (Arsi and East Shewa Zones) (Fig. 6).

#### Wasting

**Fig. 7:**
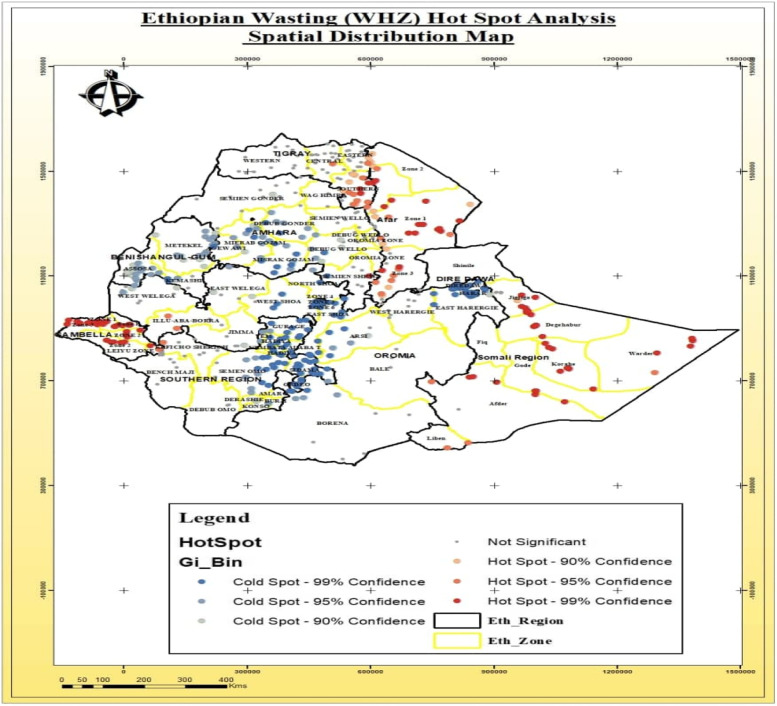
Spatial distribution of Hotspots and Cold spots areas of wasting among under-five in Ethiopia

The hotspot area distribution of wasting among those under five in Ethiopia were detected the in Somali Region (Afder, Gode, Korahe Zones), Afar region (Awsi Zone_and Zone 1), Gambella Region in Anuak and Mezenger zones). In contrast, cold spot areas were found in the SNNPR region (Gurage and Gedeo Zones), and some parts Oromia region (West Shoa, East Shoa, and Arsi Zones) and Diredawa (Fig. 7).

### Spatial distribution of toilet facility and source of drinking water

**Fig. 8:**
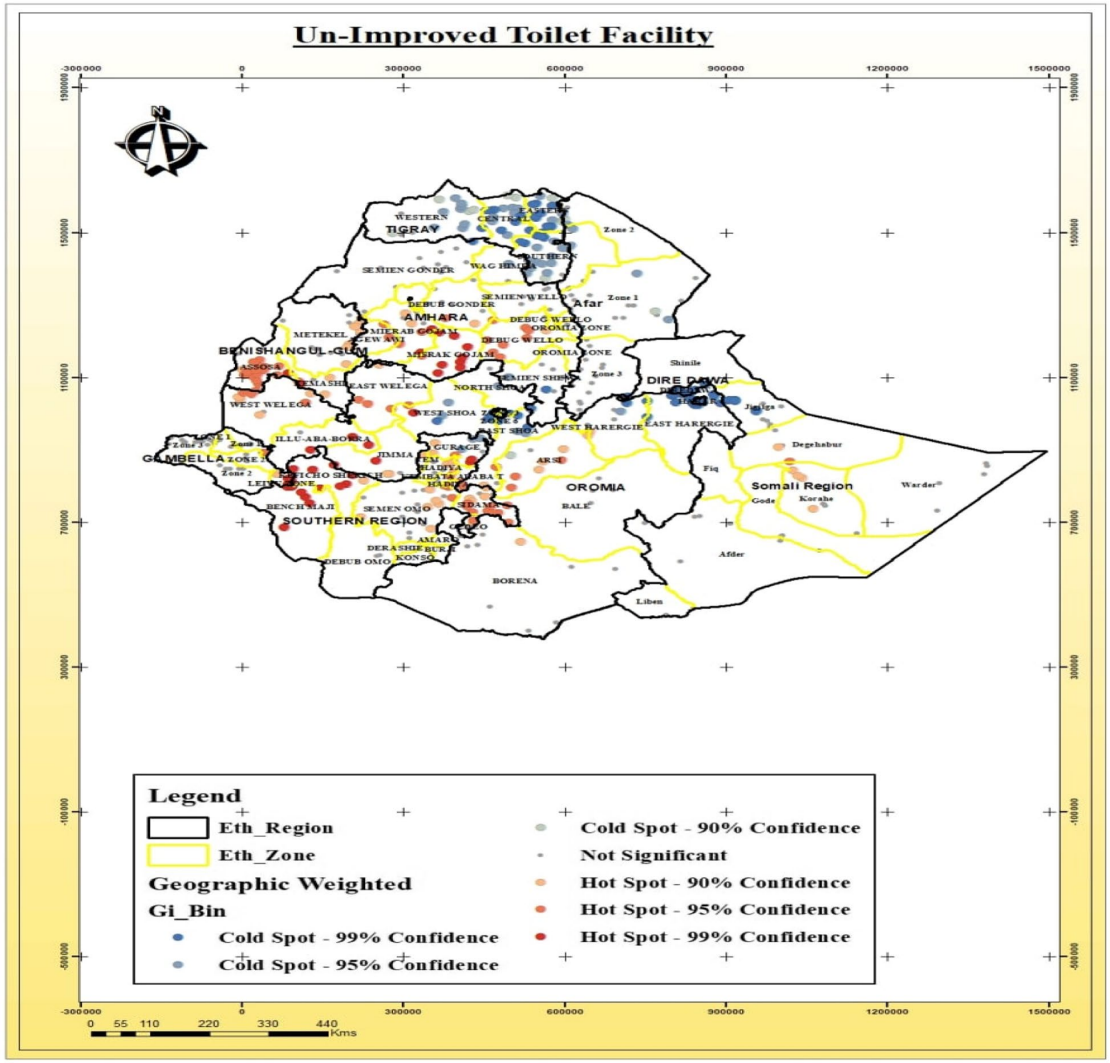
Spatial distribution of un-improved toilet facility among households in various Ethiopia regions

The spatial distribution of community-level factors like the type of toilet facility and source of drinking water has been detected in Ethiopia. These two factors had a significant association with the stunting, wasting and underweight (Table 3). Thus, it is important to identify areas where the un-improved toilet facility and unimproved source of drinking water for households were geographically located in Ethiopia as shown below (Fig. 8).

**Fig. 9:**
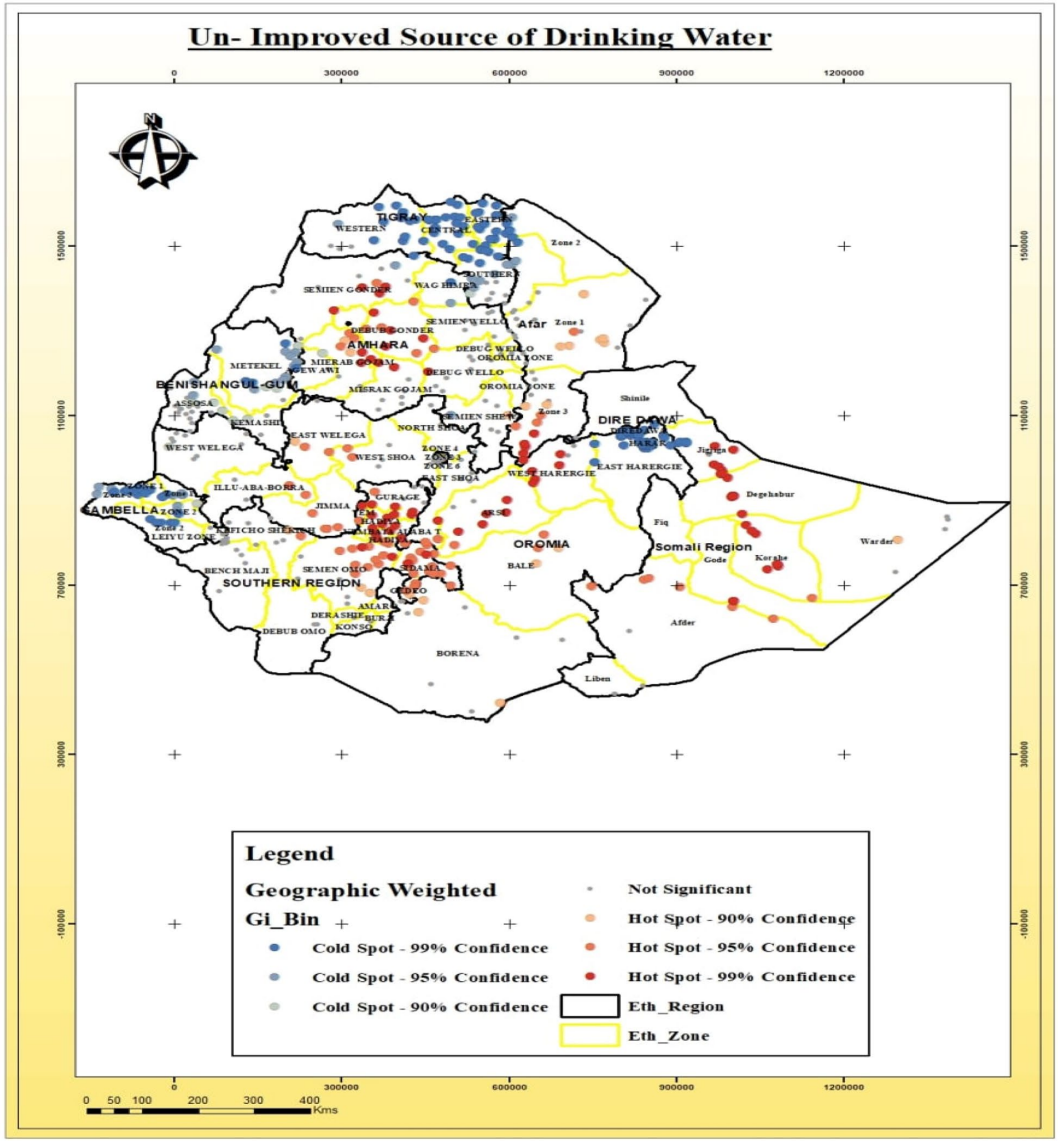
Spatial distribution of un-improved drinking water sources among households in the various Ethiopia Regions

The spatial distribution of access to the toilet facility among the households was detected across and within regions of Ethiopia. Un-improved toilet facility was observed among households residing in some zones of the Amhara region (North Gondar, South Gondar, West Gojjam Zone, Awi Zone, and South Wollo Zones), parts of the Benshangul-gumuz region, especially Asosa and Kemashi Zones, parts of the Somali region around Degehabur Zone, in the Somali region specifically in Jijjiga, Korahe, and Degehabur Zones and in Afar region, especially in Gabi Zone_Zone 3 (Fig. 9).

## Discussions

This study was aimed to explore the spatial distribution of malnutrition (stunting, wasting and underweight) in children under-five in Ethiopia and to identify determinant factors. The Moran’s I spatial autocorrelation statistics points that there was spatial dependencies, the geographical gradients and the clear spatial pattern of malnutrition among under-five between and within regions and zones of Ethiopia.

Mothers’ and father’ educational level had a significant association with the under-five malnutrition. Findings from the similar studies have confirmed that the family educational status have a significant association with stunting, wasting and under five in children under-five [32]. In the present study, the age of the child was significantly associated with the stunting, wasting and being underweight. The studies conducted in Bangladesh, Madagascar, and Malawi had also reported that due to the inappropriate and late introduction of low nutritional quality supplementary food and a large portion of guardians in rural areas are ignoring to meet their children’s optimal food requirements as the age of the child increases [33–35]. The Body Mass Index (BMI) of the mother of a child had significant association with malnutrition of a child under-five. Other studies had also confirmed that a change in the effect of maternal BMI had significant effect on under-five malnutrition, whereby children whose mother was obese were more likely to be stunted, wasted, and underweight [36]. Children who had longer breast feeding durations were less likely malnourished (stunted, wasted and being underweight) compared to children who had shorter breast feeding duration. As stated in the other research findings, shorter breast feeding duration (below six months) could impact the nutrition well-being of the a child and also prolonged breastfeeding can enable eye-to-eye contact, physical closeness, and emotional bonding, which are essential for optimal child growth and development [37].

The current study has tried to identify significantly hotspot areas of stunting, wasting and underweight in Ethiopia. The most stunted under five children were found in West Gojjam and Awi Zone of Amhara region, Metekel Zone of Benishangul-gumuz region, and in the Somali region. Similar findings were reported by the study conducted in Ghana which indicates that stunting was randomly distributed in the areas under consideration [38]. However, due to the some intervention strategies for stunting in some selected clusters and due to the variation from temporal and environmental factors that may trigger the clustering of cases in a certain geographical areas, findings from current study was contradicting with the research findings reported from Rwanda [39] and Sub-Saharan Africa [40]. The spatial distribution of underweight was also clustered across the region and varies within the zones in the specific region. The similar findings was reported by a study conducted in Ethiopia that has detected that the Afar and Somali regions were the hotspot areas for underweight [41]. But, the previous study has reported that Fanti Rasu zone of Afar region, Metekel zone and Assosa administrative zones of Benishangul-gumuz region were identified as cold spot area for underweight in children under-five [42].

The statistically significant association was estimated between community level factors (toilet facility and sources of drinking water) for households in different regions of Ethiopia. There was significantly different access and distribution of toilet facilities and source of drinking water across regions and the zones within the same region. Unimproved toilet facility and un-improved sources of drinking water were one of the potential sources of spatial variation for the areas detected as hotspots for stunting, wasting, and underweight. Similar findings from a study conducted in Kenya [43] have confirmed that in the context of unimproved toilets, on most occasions, households having unimproved toilets facility could be linked with the hygiene issues or poor wealth status and this, in turn, could influence the nutritional status of the under-five.

The findings from the various studies have been suggested the alternative and ground breaking approached on how to improve the under-five child care and also how to reduce the burdens of malnutrition in developing countries including Ethiopia. However, the magnitude of the problem looks not yet given the right and quick response to reduce the size of the burden. Thus, the authors want to suggest the following policy alternatives and also would like to underline on some issues to be focused by the Ethiopian government and the other stakeholders. The government should have to develop evidence-based guidelines based on robust scientific and ethical frameworks; align and advocating for nutrition priorities and policies on the ground; facilitate adoption of nutrition related guidelines and should focus on implementation of effective measures; should have to have a regular monitoring and evaluation with special attention on the malnutrition policy and program implementation. Ministry of Health (MOH) of Ethiopian and other concerned stakeholders should have to make groundbreaking interventions in those hotspots regions as well as the Zones. The government should have to follow alternative strategies to improve the maternal and child care facility, provide improved living standard for the households who had unimproved drinking water access and toilet facility and increase the formal education coverage in the Zones and regions where there was hotspot areas for malnutrition. Specifically, the special interventions strategy should be set to reduce the burden of geographically clustered under- five malnutrition problem in Ethiopia.

### Limitations of the study

The current study was aimed to estimate the spatial distribution and prevalence of malnutrition in Ethiopia taking into account variations within and between regions. The secondary data from EDHS, 2016, which is older survey data used in the analysis to estimate association between variables expected contribute to the malnutrition among under-five. However, no causation analysis is done. Some of the important malnutrition predictors are not available in the EDHS, 2016 data. Variables with missing responses were removed during sample size determination and missing data analysis approach is not used in the current study.

## Conclusions

The main aim of current study was to analyze spatial distributions, detect hot spot and cold spot areas, explore whether there was random clustering and to identify determinant factors of malnutrition (stunting, wasting, and underweight) in children under-five in Ethiopia. There was covenant and non-random spatial variation of stunting, wasting, and underweight in children under-five across and within the regions of Ethiopia. The spatial clusters of stunting, wasting and underweight was found in geographical pocket areas where there was the high distribution of un-improved water and un-improved toilet facility among households across and within the regions. The highest prevalence of stunting, wasting and underweight was detected in the regions where there was poor healthcare facilities and less socioeconomic status (Benshagul region, Somali region: Afder, Gode, and Korahe Zone, and Afar region: Awsi Zone).

## Supplementary Information

**Additional file 1.**

## Data Availability

We have accessed the publically available data based on the available data access permission set by the agency under official web page (https://dhsprogram.com/data/available-datasets.cfm).
