# Supplementary material for: Spatial distribution and determinants of stunting, wasting and underweight in children under-five in Ethiopia
Source: BMC Public Health. 2023 Apr 4;23:641. doi: 10.1186/s12889-023-15488-z (PMC10071774; doi:10.1186/s12889-023-15488-z)
Supplement: Supplementary file 1 — Additional file 1. [file 12889_2023_15488_MOESM1_ESM.docx]

Supplementary Table 1: Description of Independent Variables and Respective Cods (EDHS, 2016)

| **Variables** | **Code** |
| --- | --- |
| Sex of child | 0 = Male, 1 = Female |
| Birth order | 0 = First, 1 = 2-4, 2 = >4 |
| Birth size | 0 = Large,1 = Medium, 2 = Small, |
| Number of children under five in the household | 0= No, 1 = 1-2, 2 = >= 3 |
| Mother's educational level | 0 = No education, 1 = Primary, 2 = Secondary, 3 = Higher |
| Preceding birth interval (in months) | 0 = < 24, 1 = >= 24 |
| Mother’s Body Mass Index (BMI) | 0 = Thin(<18), 1= Normal(18-24.9), 2 = Obese (>= 25) |
| Mother’s occupational status | 0 = No occupation, 1 = had occupation |
| Father’s educational level | 0 = No education, 1 = Primary level, 2 = Secondary level, 3 = Higher (above secondary level) |
| Child’s breast feeding duration | 0 = Ever breastfed(not currently breastfeeding), 1= Never breastfed,  1 = Still breastfeeding, |
| Place of residence | 0 = Urban, 1 = Rural |
| Region | 0 = Tigray, 1= Afar, 2 = Amhara, 3 = Oromia, 4 = Somali,  5 = Benishangul, 6 = SNNPR, 7 = Gambela, 8 = Harari, 9 = Addis Adaba, 10 = Dire Dawa |
| Source of drinking water | 0 = Improved source  1 = Un-Improved source |
| Toilet facility | 0 = Improved toilet/sanitation  1 = Un-Improved toilet/Sanitation |
| Sex of household head | 0 = Male, 1 = Female |
| Type of cooking fuel | 0 = Modern fuel  1= Traditional fuel |
| Wealth index of Mothers | 0 = Poor, 1 = Medium, 2 = Rich |
| Marital Status of mother | 0 = Never in union, 1 = Married/Living With Partner,  2 = Widowed/Divorced/ Separated |
| Age of mothers at first birth (in years) | 0 = <18, 1 = 18-25, 2 = 26-35, 3 = 36-45 |
| Age of child (in months) | 0 = < 6, 1 = 6-12, 2 = 13-24, 3 = 25-36,  4 = 37-48, 5 = 49-59 |

| **Names of Zones in Ethiopian Regions** | | |  | | | |
| --- | --- | --- | --- | --- | --- | --- |
| Tigray region (0) | Afar region (1) | Amhara region (2) | Oromia region (3) | | | Somali region (4) |
| Zones | Zones | Zones | Zones | | | Zones |
| 0 = North West Tigray  1 = Central Tigray  2 = East Tigray  3 = South Tigray  4 = West Tigray  5 = Mekele Special Zone | 0 = Awsi Rasu  1 = Fanti Rasu  2 = Gabi Rasu  3 = Hari Rasu  4 = Kilbet Rasu | 0 = North Gondar  1= South Gondar  2 = North Wollo  3= South Wollo  4 = North Shewa  5 = East Gojjam  6 = West Gojjam  7 = Wag Hemra  8 = Awi  9 = Oromia-Special Zone  10 = Bahirdar (special zone) | 0 = West Wellega  1 = Kellem Wellega  2 = East Wellega  3 = Horo Guduru Wellega  4 = Illu Ababora  5 = Jimma  6 = West Shewa  7 = South West Shewa  8 = North Shewa  9 = East Shewa | | 10 = Arsi  11= West Arsi  12 = West Hararghe  13 = East Hararge  14 = Bale  15 = Borena  16 = Guji  17 = Adama (special zone) | 0 = Shinile  1 = Jijjiga  2 = Degehabur  3 = Fiq  4 = Korahe  5 = Gode  6 = Werder  7 = Afder  8 = Liben |
| **Benishangul -gumuz region (5)** | **SNNPR region (6)** | | **Gambela region (7)** | **Harari** **region (8)** | **Addis Ababa(10)** | **Dire Dawa(11)** |
| Zones | Zones | | Zones |  |  |  |
| 0 = Metekel  1 = Asosa  2 = Kemashi | 0 = Gurage  1= Silt’e  2 = Hadiya  3 = Kembata Tambaro  4 = Sidama  5 = Gedeo  6 = Wolayita | 7 = Dawro  8 = Gamo Gofa  9 = South Omo  10 = Sheka  11= Keffa  12 = Benchi Maji | 0 = Anuak  1 = Nuer  2 = Mezenger | - | - | - |
